# BENEFICIAL EFFECTS OF A PLASMA FRACTION ON NEUROGENESIS FOR AGE-RELATED COGNITIVE DISORDERS

**DOI:** 10.1093/geroni/igz038.345

**Published:** 2019-11-08

**Authors:** Ian Gallager, Viktoria Kheifets, S Sakura Minami, Eva Czirr, scott lohr, nina huber, meghan Campbell, Steven P Braithwaite

**Affiliations:** Alkahest Inc., San Carlos, California, United States

## Abstract

The process of aging is multifactorial and therefore single interventions may be unlikely to attenuate the myriad pathologies. Plasma contains many beneficial factors which have been shown in animal models to ameliorate multiple age-related deficits across varied organ systems, including the brain. We demonstrated that human plasma from young 18-22-year-old donors reverses age-related cognitive decline and enhances hippocampal neurogenesis and cell survival in aged immunocompromised mice, while plasma from aged individuals (62-68 years old) has detrimental effects in young mice. Utilizing cell-based assays we identified a human plasma fraction (PF) which enhanced neuronal outgrowth and synaptic connectivity. We demonstrate that PF administration provides benefits beyond those observed with whole plasma treatment, resulting in decreased brain inflammation, increased synaptic density and neuronal activation. We evaluated longitudinal treatment of PF and observed no depletion in stem cell populations while maintaining an enhanced level of neurogenesis. We examined PF in an α-synuclein mouse model of Parkinson’s disease, where treatment significantly reversed functional, inflammatory, and neuronal deficits. To further our understanding of interplay between multiple mechanisms inducing neurogenesis, we examined the effect of exercise on PF treated mice and observed a synergistic increase in neurogenesis. Proteomic analysis of mouse plasma following PF treatment demonstrates differential protein levels compared to running or the combination of running and PF, suggesting that PF is mechanistically different from the effect of running. In summary, we demonstrate that PF is a multifactorial and multimodal, clinically-relevant, intervention for the treatment of global changes induced by aging.

